# The fiduciary obligation of the physician-researcher in phase IV trials

**DOI:** 10.1186/1472-6939-15-11

**Published:** 2014-02-07

**Authors:** Rosemarie DLC Bernabe, Ghislaine JMW van Thiel, Jan AM Raaijmakers, Johannes JM van Delden

**Affiliations:** 1 Julius Center for Health Sciences and Primary Care, Utrecht University Medical Center, Heidelberglaan 100, Utrecht 3584 CX, The Netherlands; 2 GlaxoSmithKline, Zeist, The Netherlands

**Keywords:** Phase IV, Fiduciary obligation, Interventional trials, Physician-researchers

## Abstract

**Background:**

In this manuscript, we argue that within the context of phase IV, physician-researchers retain their fiduciary obligation to treat the patient-participants.

**Discussion:**

We first clarify why the perspective that research ethics ought to be differentiated from clinical ethics is not applicable in phase IV, and therefore, why therapeutic orientation is most convivial in this phase. Next, assuming that ethics guidelines may be representative of common morality, we show that ethics guidelines see physician-researchers primarily as physicians and only secondarily as researchers. We then elaborate on what a fiduciary obligation is and how some of the obligations are default duties. Lastly, we look at the fiduciary obligation of the physician-researcher in phase IV interventional trials.

**Conclusion:**

The fiduciary obligation to treat is not as easily waived as in earlier trials. Assuming the entwinement of research and practice in phase IV, physician-researchers, in collaboration with other researchers, investigators, and research ethics committees, should ensure that in terms of study design, methodology, and research practice, the therapeutic value of the research to the patient-participants is not diminished.

## Background

A more rigorous and scientifically robust phase IV within the drug development system [1] is important both for the continuous appraisal of the benefit-risk profile of a drug and for the evaluation of the drug’s economic value (for reimbursement purposes, for example). In the case of the former, the continuous appraisal of a drug’s benefit-risk profile is highlighted by the present drive towards a shorter but more efficient pre-authorization drug development phase [2,3] and the concomitant move towards progressive authorization, that is, authorization that allows earlier but limited release of a drug and license expansion is dependent on new data [4]. On top of that, there is increased attention on phase IV studies required by the FDA due to safety signs that may affect the benefit-risk profile of a drug [5,6].

By phase IV, we refer to “all studies (other than routine surveillance) performed after drug approval and related to the approved indication” such as “drug-drug interaction studies, dose–response or safety studies and studies designed to support use under the approved indication” [7] as well as studies to obtain health economic data. These studies are usually “larger, less technically complicated than pre-registration studies, have fewer inclusion/exclusion criteria and are more likely to include subjective or qualitative end points” [8]. Also, these studies are meant to gather real-world data for the purpose of informing clinical decisions [9,10]. As such, many or most phase IV studies occur within the doctor’s clinic.

Granted the fact that not all safety and efficacy issues are known at the time of approval, it is also a given fact that upon authorization, drugs are declared to have proven safety, efficacy, and quality, and as such, it is reasonable for patients and medical practitioners to have expectations of benefit from these drugs. In a phase IV study, therapy and research are necessarily intertwined due to the tension between the reasonable expectation of benefit and the knowledge gaps in drug safety and efficacy. Social policies such as coverage with evidence development assume this entwinement by making access to therapy dependent on research participation. This entwinement is highlighted by the fact that these studies occur within the doctor’s clinic, and thus the physician-researcher conflict is especially perceived in phase IV.

In this paper, we shall argue that *since phase IV trials are by nature and purpose closer to practice than the other phases of drug development, physician-researchers are primarily physicians and secondarily researchers whose fiduciary obligation to their patient-participants remains, although some aspects of this obligation may be waived.* When we speak of physicians, we refer specifically and narrowly to treating physicians and not to career researchers who happen to have a medical degree. By career researchers, we refer to those who do not have patients themselves; rather, they are full-fledged researchers who might be working for CROs or pharmaceutical companies. Further, we shall limit ourselves to phase IV trials that are interventional, i.e., trials where the therapy that the patient-participant receives is dictated by the protocol; the physician’s prescription is related to the patient-participant’s inclusion in the trial; and additional diagnostic or monitoring procedures are necessary [11].

To achieve our aim, we shall first show that physician-researchers are primary physicians and secondarily researchers by looking at relevant ethics guidelines. Next, we shall discuss what the latter means by situating our discussion within that of fiduciary obligation. Within this discussion of fiduciary obligation, it is necessary to briefly take a step back and show that the physician-patient relationship is fiduciary in nature, and that some aspects of this obligation may be waived for the purpose of research. Lastly, we revisit phase IV and demonstrate the implications of our main thesis.

### The conflict: phase IV research and therapeutic obligation

#### Two approaches to clinical research ethics dilemmas

Dilemmas in clinical research ethics are usually approached by either A.) stating that research and practice are different and hence the ethical requirements of research ought to be differentiated from the requirements of practice; or, B.) by viewing research as essentially related to practice. These two approaches are best represented by the discussion in the literature on therapeutic orientation [12-16]. By therapeutic orientation, we refer to the inclination or the mindset where research is seen in terms of therapeutic morality [12], i.e., the mindset that does not fully separate or fully distinguish the following:

…“practice” refers to interventions that are designed solely to enhance the well-being of an individual patient or client and that have a reasonable expectation of success…. By contrast, the term “research” designates an activity designed to test a hypothesis, permit conclusions to be drawn, and thereby to develop or contribute to generalizable knowledge… [17].

F. Miller and Rosenstein [12] and Appelbaum and Lidz [18] may be representative of perspective A on the practice-research distinction. For them, the difference between research and practice in terms of aims and relationships is ethically significant: research aims to “produce generalizable knowledge” while practice aims to “provide individual patients with optimal care”. Hence, therapeutic orientation ought to be seen as a problem that must be solved in research ethics. On the other hand, Anderson [19], Freedman [20], Comoretto [15], and Lemmens and P. Miller [21] are some examples of thinkers who represent perspective B. For Anderson, for example, research and practice are epistemologically linked since practice provides methodological constraints to research [19]. In terms of therapeutic orientation, removing it from research is an approach [21] that unnaturally separates research from its goal, i.e., therapeutic benefit.

Depending on which perspective we take, we can provide different responses to our main question. In this paper, we relate more to perspective B and we shall do so by putting this therapeutic orientation within the discussion of the fiduciary obligation of physician-researchers. But before we explicate our point, some words are in order on the entwinement of research and practice in phase IV.

#### Why perspective A is not applicable to the physician-researcher dilemma in phase IV

Phase IV is a peculiar phase among the other phases of drug development precisely because of the marketing authorization of the drug being studied within this phase. Thus, unlike earlier phases, a comparative phase IV trial refers to the comparison of two authorized drugs. Authorization means that it is logical to expect a two-armed trial between a trial drug and a non-placebo comparator to have reasonable levels of safety and effectiveness. This is a noteworthy difference between phase IV and the other phases where safety and efficacy cannot yet be reasonably expected^a^. This means that to a certain extent, any phase IV trial that excludes a placebo arm is naturally and essentially therapeutic.

A phase IV interventional study is therapeutic not only because, in the absence of placebo, the drugs involved are licensed drugs; as mentioned above, the purpose of such trials is to inform clinical decisions. In an earlier study, we have elaborated on informing a decision as follows:

…a phase IV trial should aim at “informing a clinical decision.” We defined “informing a decision” to refer to clinically relevant differences that would allow physicians to reasonably choose one drug over another… However, these clinically relevant differences also matter in the decision-making processes of the other stakeholders such as the regulators, patient groups, pharmaceutical industry, and third party payers. The importance of these clinically relevant differences is illustrated by the emergence of relative effectiveness as an important issue in the post-authorization stage… Ultimately, the aim of relative effectiveness assessment is “to compare healthcare interventions in practice in order to classify them according to their practical therapeutic value” [9].

Thus, a phase IV study basically aims to effectively and positively affect clinical decision-making by gathering real-life data as much as possible

As a consequence of the therapeutic nature of phase IV whose goal is to gather (real-life) data that informs clinical decisions, the common setting for these usually large and less technically complicated trials is the physician’s clinic. We wish to note that though earlier phases are done in clinics/hospitals as well, there is still a difference: earlier phases are done in clinics because these are convenient research centers where necessary medical tests and procedures may be done. Phase IV trials, on the other hand, are done in clinics precisely because these trials are meant to gather data about a drug once it is sent out to the world. Simply, phase IV trials are intimate with practice, i.e., therapy is not just a “common effect” of a phase IV interventional study; rather, it ought to be a necessary consideration in its design and execution.

Since by nature and purpose phase IV trials are closer to practice than the other phases, perspective A, which demands two separate ethics for research and practice, is simply not applicable for this phase. In phase IV, research and practice are intertwined. In what follows, we shall discuss that physician-researchers are primarily physicians and only secondarily researchers; after which, we shall elaborate on what this means by putting this discussion within the context of fiduciary obligation.

### Physician-researchers are primarily physicians

Physician-researchers are primarily physicians who are engaged in research; they are not dissociative identity professionals who struggle between being fully a physician and being fully a researcher. By looking at some medical ethics guidelines for physicians, we get a better grasp of this. Admittedly, we are not *arguing* for this point; rather, we are demonstrating that based on the ethics codes of some medical associations, it is the predominant perception that physician-researchers are primarily physicians who are doing research.

The 2013 Helsinki Declaration is unambiguous in terms of the priorities of physicians involved in research (refer to Table 1). Since the Helsinki Declaration is a document that was ratified with the participation of delegates from its constituent member countries, and organizations such as the International Conference on Harmonisation [22] and the European Medicines Agency [23] have since acknowledged the authority of this declaration without issuing any divergence or disagreement with it, the moral ascendancy of this declaration over its constituent member countries is almost without question. This is the closest we can get to common morality in the absence of a survey of all the existing national codes of ethics.

**Table 1:** Ethical codes of some medical associations that state what the priority is of the physician-researcher when doing research

**Medical association**	**Statement in the medical ethics guideline on physician priority in research**
World Medical Association [24]	Declaration of Helsinki:
	3. The Declaration of Geneva of the WMA binds the physician with the words, *“The health of my patient will be my first consideration,”* and the International Code of Medical Ethics declares that, *“A physician shall act in the patient’s best interest when providing medical care.”*
	4. It is the duty of the physician to promote and safeguard the health, well-being and rights of patients, including those who are involved in medical research. The physician’s knowledge and conscience are dedicated to the fulfilment of this duty.
	8. While the primary purpose of medical research is to generate new knowledge*, this goal can never take precedence over the rights and interests of individual research subjects.*
Australian Medical Association [25]	AMA Code of Ethics:
	1.2 Clinical Research
	c. Recognise that considerations relating to *the well-being of individual participants in research take precedence* over the interests of science or society.
UK General Medical Council [26]	Good Medical Practice (Research):
	71. If you are involved in designing, organising or carrying out research, you must:
	a. *put the protection of the participants’ interests first*

In agreement with and as a support to what is stated in the Helsinki Declaration, the Australian Medical Association’s and the UK General Medical Council’s codes of medical ethics provide explicit and straightforward statements on physicians’ priorities when involved in research (refer to Table 1). We wish to note that since we have not done an exhaustive comparison of the various national codes of medical ethics, it is possible that some national codes of medical ethics would disagree with those of the UK and Australia or even with Helsinki. However, since we assume the primacy of Helsinki, and since we used the UK and Australian codes as auxiliaries, we believe our stance will not be substantially affected by a potential disagreement between national codes.

Hence, it seems at the very least agreed upon by the international medical community (and the medical communities of the two countries concerned) that these are codes of ethics *for physicians who may be doing research*. Hence, physician-researchers are not individuals with two contrasting and separate hats: that of the physician and that of the researcher. Rather, they are physicians who must follow certain guidelines when involved in research. Second, these ethics codes explicitly state that in the event that a physician engages herself/himself in research, the priority is clear: the well-being/health/rights/interest/protection of the patient-participant. Granted that there may be variations on how to interpret words like “well-being” or “interest”, it is still without doubt that these various codes require the physician to put the patient first over research interests.

### The fiduciary obligation of physicians and the possibility of waiver

In the succeeding sections, our ultimate aim is to provide clarification of what it means for physicians, as fiduciaries, to put the patient-participant first within the context of phase IV. However, before proceeding, we need to take a few steps back: first, it is necessary to discuss what we mean by a fiduciary relationship; second, describe the physician-patient relationship as a fiduciary relationship; and third, discuss the possibilities of waiver of the fiduciary obligation for purposes of research.

#### Fiduciary relationships

A fiduciary relationship is a service relationship that is meant for the provision of a service that public policy encourages [27]. In a fiduciary relationship, two or more persons are involved: the fiduciary, the entrustor, and in some instances, the beneficiary who may be distinct from the entrustor such as the case of trustors-trustees-beneficiaries. Relationships such as the following are considered fiduciary: solicitor-client, director-corporation, trustee-trustor, and of course, physician-patient [27,28]. A fiduciary relationship is distinct from a contractual relationship due to the power imbalance between the fiduciary and the entrustor. In a contract relation, the two contracting parties are considered as independent; in a fiduciary relationship, the fiduciary is entrusted with power to enable her/him to provide a specific service to the entrustor. Precisely because of this power and the service that the fiduciary provides through this power, the entrustor is to a certain extent necessarily dependent on the fiduciary [29].

Traditionally, the duties of the fiduciary may be categorized into two: duty of loyalty and duty of care. The duty of loyalty refers to the broad category of preventative duties that protect the entrustor’s right to honesty, and hence, the prevention of the fiduciary from using power without authorization [27]. To uphold this broad duty, fiduciaries may be required, for example, to account for entrusted assets, to not compete with the entrustor within the service area concerned, and not to create situations in which the fiduciary may have a conflict of interest that may compromise the entrustor [27].

The duty of care, on the other hand, addresses the entrustor’s right to receive quality service (in terms of reasonable care and skill) from the fiduciary [27].

Without needing to go to details, it is important to note that the degree of strictness of the fiduciary duties varies from one type of fiduciary to another and these variations depend on the type of power entrusted to them, the availability of “monitoring and controls” that entrustors have over the fiduciaries, the cost of using these monitors and controls over the fiduciaries, the gravity of the risk with which entrustors are exposed to due to the imposition of power to the fiduciary, and the lack of alternatives to protect entrustors from these risks [27].

#### Physician-patient relationship as fiduciary relationship

That physicians are fiduciaries to their patients is best expressed in cases such as *Norberg v. Wynrib.* In the latter case, for example, the Canadian Supreme Court unequivocally characterized physician-patient relationship as *fundamentally* fiduciary in nature [30]. As fiduciaries, without discounting the patients’ right and capacity for self-determination, physicians have duties of loyalty and care towards their patients. In terms of the duty of loyalty, the demands of which we have outlined above, in compliance with their duty to account for “entrusted assets”, physicians are required to protect the patient’s privacy by for example safeguarding the patient’s information. They should also not directly market and sell drugs to their patients, as this puts their interest in direct conflict with that of their patients’. Engaging in “inappropriate sexual relationship or committing sexual misconduct” [31] is another example where physicians breach their duty of not letting their personal interest conflict with their patients’ [32]. The breach of the duty of loyalty in all of these cases exemplifies abuse of the power vested on the physician. We shall see in a short while that though engaging in research also qualify as a conflict of interest, there are procedures that allow for the waiving of the duty to not engage in activities that conflict with the patients’ best interest.

The duty of care, on the other hand, refers to the duty of physicians to “exercise reasonable care, diligence and skill in the exercise of their discretionary power (e.g., clinical judgment in the diagnosis and treatment of a patient)” [32]. This may refer, for example, to physicians following GCP protocols, requesting the necessary diagnostic tests before providing a diagnosis, engaging the patient in the treatment through shared decision making and personalized care, providing balanced treatment options and recommendations based on the patient’s individualized condition, providing treatment follow-ups, among others.

#### Possibilities of waiver of some of the fiduciary obligations for purposes of research

In some instances, fiduciary duties may be considered as default rules, i.e., rules that “apply unless otherwise agreed” [33]. Default rules may be waived through some sort of agreement and procedure.

According to Frankel, most fiduciary obligations are default obligations [34]. As such, it is possible for some fiduciary obligations not only to be waived, but also to be replaced by a contract, i.e., fiduciaries may propose to their beneficiaries that some of their responsibilities be waived and contracted. However, due to the dependence of entrustors on beneficiaries, waiving may only be possible if the following procedures are followed:

Fiduciaries must put entrustors on notice that, regarding the specified transaction, entrustors are on their own; entrustors must have legal capacity to enter into bargains with their fiduciaries as independent parties; to enable entrustors to make informed decisions, fiduciaries must provide them with information regarding the transaction… [27].

The above-mentioned procedure is exactly the principle behind informed consent in research: patients are informed, and hence put on notice, that once they sign the form, certain obligations of the physicians are waived or compromised due to research; in the process of obtaining informed consent, the patient’s capacity to consent must be ascertained; lastly, in informing the patient about the research, information sheets (or other similar materials) are provided and explained to make sure that all the necessary research procedures and repercussions are explained.

In the literature, there are discussions whether the physician-researcher has fiduciary obligations to patient-participants [35-37]. We think the literature has contributed greatly in expounding on the negative and positive obligations of researchers towards research participants*. However, based on the foregoing discussion, we think it is unnecessary to labor whether the physician, as a researcher, has a fiduciary obligation towards the patient-participant. It makes much more sense to view the physician-researcher as someone whose primary fiduciary obligation is to take care of the well-being of the patient, but in instances of research, she/he waives some of these obligations through the informed consent procedure.*

In research, some obligations are waived such as the obligation towards personalized care, or the obligation of loyalty not to conflict with the patient’s best interest. The patient-participant will be placed in a situation where treatment will be based on the protocol and not only on the evaluation of the patient’s individualized condition. However, as a natural fiduciary, the physician-researcher retains her/his unwaived obligations such as the obligation to deliberate on the patient’s situation, and hence, decide whether it is still reasonable for the patient to be part of the trial or whether, for the patient’s interest, the patient ought to be removed from the trial. Or, in the event that the physician-researcher is involved in the design of the trial, she/he must “put the protection of the participants’ interests first” [26] by making sure that proper safety procedures are present and that risks are acceptable to and for the patient-participants. Indeed, Helsinki article 9 lists the physicians’ (fiduciary) duties that are retained in a trial:

It is the duty of physicians who are involved in medical research to protect the life, health, dignity, integrity, right to self-determination, privacy, and confidentiality of personal information of research subjects. The responsibility for the protection of research subjects must always rest with the physician or other health care professionals and never with the research subjects, even though they have given consent [24].

Hence, not all fiduciary duties are default duties. Frankel mentions three instances when fiduciary obligations are “mandatory”: when leveling the playing field for fiduciaries; when protecting the fundamental tenets of society; and when providing paternalistic protections [34]. For our purposes, we shall call these generic mandatory fiduciary obligations. By generic, we mean that these obligations apply to all physician-researchers who are involved in *any* clinical trial. It will be best to briefly illustrate how some of the restrictions in ethics guidelines that are very much familiar to us may be rooted or contextualized within these generic mandatory fiduciary obligations.

##### 

**Leveling the playing field** The retained obligations in Helsinki article 9 as we saw above provide for a level playing field. This means that these retained obligations ensure that the quality of services is not “less-than-acceptable” [34] in *all* clinical trials. The field is not only more or less level for patient-participants but for the physician-researchers as fiduciaries as well.

##### 

**Protecting society’s fundamental tenets** A physician may also not engage her/himself in a research with unacceptably high risks or with research that is methodologically faulty because researches such as these go against the fundamental tenet of society of protecting its citizens against undue harm. As somewhat an articulation of a prohibition on physicians from involving themselves in trials with unacceptable risk, Helsinki article 18 states the following: “Physicians may not be involved in a research study involving human subjects unless they are confident that the risks have been adequately assessed and can be satisfactorily managed” [24]. In the same manner, Helsinki article 21 also stipulates that a trial must in all instances be scientifically sound; as such, physicians may not engage themselves in researches that are methodologically faulty or are only scantly scientific but are cover-ups for other purposes such as marketing and promotion.

##### 

**Providing paternalistic protections** In instances of patient-participant incompetence, paternalistic protections require physician-researchers, for example, to deal with both the patient-participant and her/his legally authorized representative and to clearly explain to them which aspects of “care are related to the research” (Helsinki article 31).

With these points clarified, we can now return to our main context, i.e., phase IV.

### The physician-researcher within the context of phase IV

We earlier saw that phase IV trials are intimately linked with practice such that the therapeutic expectation of the patient-participant is not something that needs vigorous dispelling: with the exception of placebo arms, safety and effectiveness ought to be reasonably expected; in addition, this therapeutic expectation from patient-participants, and maybe even of the physician-researchers, is part of real life, the setting from which phase IV trials aim to gather evidence from to eventually inform clinical decisions. With this in mind, and given our earlier discussions of the guidelines and fiduciary obligation, we can with confidence say that *within the context of post-authorization trials, physician-researchers put the interest of the patient-participants first by being fiduciaries who retain their therapeutic obligation to their patient-participants.* Hence, in this phase, on top of the generic mandatory obligations of physician-researchers as fiduciaries we discussed above, the fiduciary obligation of the physician to treat (i.e., her/his therapeutic obligation) is not easily waived as in other phases^b^.

If the fiduciary obligation to treat of the physician-researcher is not as easily waived in phase IV, this would have concrete repercussions on the design, methodology, and manner of conducting such trials. This therapeutic obligation would for example affect the use of placebo and of noninferiority trial design in this phase. Before we spell out what some of these repercussions may be, we need a caveat: even though the end-provider of this therapeutic obligation in phase IV is the physician-researcher, the responsibility of safeguarding the therapeutic aspect of phase IV cannot lie on the physician alone. Physician-researchers, investigators, and research ethics committees should all allow for this environment where this therapeutic obligation of the physician is upheld. Concretely, this may mean their cooperation to fulfill the following:

1.) as much as possible, placebo is not used^c^;

2.) nonblinded studies are preferred over blinded ones to allow the physician-researcher to discuss the ramifications, effects, and side-effects of the drug to the patient-participant^d^;

3.) superiority trials are preferred over noninferiority trials due to the clinical value of the previous over the latter^e^;

4.) trial methodology and design should consider the therapeutic value of the trial to the patient-participant;

5.) as much as reasonably possible, with the exception of the trial drug, the physician-researcher should be allowed to prescribe other drugs and request necessary diagnostic tests for therapy purposes.

The deviation from any of these points should be reasonably defended by the study sponsor to the ethics committee, and the ethics committee should find substantive justification for the said deviation.

## Conclusion

Physicians are the fiduciaries of patients and as such, they ought to put the interest of the patient first. In some instances, the waiving of some of the obligations of the physicians to the patients is possible, as in the case of research. However, fiduciary obligations are not always default obligations. In phase IV where research is more intimately related to practice, the physician-researcher ought to lean more towards practice than to research. Hence, in this phase, Concretely, this means that physician-researchers (in collaboration with other researchers, investigators, and research ethics committees) should ensure that in terms of study design, methodology, and research practice, the therapeutic value of the research to the patient-participants is not diminished.

## Endnotes

^a^We are in no way claiming that phase IV trials are absolutely of lower risk than earlier trials for the following reasons. When we speak of phase IV interventional studies, two aspects come directly into mind: 1.) the nature and purpose of the study; 2.) the registered indication for the drug. Starting with 2, the risk should be relatively lower in phase IV studies because the information obtained in the regulatory program has been built in, at least compared to that in phases II and III. Having said that, in the early phases, a stricter set of inclusion criteria will be followed, thus avoiding a potential problem with co-morbidities and substantial age differences, for instance. Once a study in phase IV is set up to have data on a broader patient population, certain unforeseen more rare risks may increase but the overall risks cannot be expected to be higher, since the most frequent, plausible and scientifically to be expected (e.g., involvement of interactions based on the use of common pathways for degradation) are known or have been excluded by the outcome of earlier studies. Hence, it is not so much the quantity but the qualitative aspects of adverse events that are of importance and hence, we would therefore not support to offer less protection. Adding to that is off-label use and although one could say that the manufacturer cannot control such use, problems for the patients can also arise. A large interventional study has the potential to offer additional information on broader use because it reflects proper, less proper, and wrong prescribing habits, where we again cannot with confidence say that protection should be less than those in earlier trials. As such, due to these various considerations, we cannot say that phase IV interventional trials indeed have lower risks than earlier phases and hence need less protection.

^b^There may be concerns that in earlier trials, especially in phase III, medical doctors may also be willing to enroll a patient in a potentially therapeutic trial because the doctor is in equipoise as to which therapy is best, and thus, the difference in terms of fiduciary obligations waived may not be significant. We argue that in phase III, the fiduciary obligation of the physician to choose based on what is best for the patient will have to be compromised, even if theoretically, the scientific-medical community is in equipoise. In phase III, there are generally more research-interventions (such as extra blood draws or other diagnostic procedures) than in most phase IV studies, and this is a further compromise of the fiduciary obligation of the physician. Placebo is also more common in phase III. In contrast, in this section, we argue that because of the therapeutic orientation of phase IV, and hence the fiduciary obligation of the physician is less compromised, placebo as much as possible should not be used, that nonblinded studies are preferred over blinded ones, that as much as possible, physicians should be allowed to prescribe other drugs and request necessary diagnostic tests, etcetera. As such, we believe we are justified in stating that less aspects of the fiduciary obligation is waived in phase IV.

^c^Last 17 May 2013, we searched http://www.clinicaltrials.gov for clinical trials using the following restrictions: placebo, interventional, and phase 4. In total, there were 3941 studies.

^d^Last 17 May 2013, we searched http://www.clinicaltrials.gov for clinical trials using the following restrictions: blinded interventional, phase IV. In total, there were 588 studies.

^e^Last 17 May 2013, we searched http://www.clinicaltrials.gov for clinical trials with the following restrictions: noninferiority, interventional, phase IV. In total, there were 130 studies.

## Competing interests

RDLCB’s PhD project was funded by the Dutch Top Institute Pharma. JAMR works for and holds stocks in GlaxoSmithKline. JJMvD and GJMWvT have no competing interests to declare.

## Authors’ contributions

All authors were involved in the design of the manuscript. RDLCB did the research and wrote the draft and final manuscript; GJMWvT commented on the drafts, wrote parts of the manuscript, and approved the final version; JAMR commented on the drafts and approved the final version of the manuscript; JJMvD commented on the drafts and approved the final version of the manuscript. All authors read and approved the final manuscript.

## Pre-publication history

The pre-publication history for this paper can be accessed here:

http://www.biomedcentral.com/1472-6939/15/11/prepub
